# Shifting Patterns of *Aedes aegypti* Fine Scale Spatial Clustering in Iquitos, Peru

**DOI:** 10.1371/journal.pntd.0003038

**Published:** 2014-08-07

**Authors:** Genevieve LaCon, Amy C. Morrison, Helvio Astete, Steven T. Stoddard, Valerie A. Paz-Soldan, John P. Elder, Eric S. Halsey, Thomas W. Scott, Uriel Kitron, Gonzalo M. Vazquez-Prokopec

**Affiliations:** 1 Department of Environmental Sciences, Emory University, Atlanta, Georgia, United States of America; 2 Department of Entomology, University of California Davis, Davis, California, United States of America; 3 U.S. Naval Medical Research Unit No. 6, Lima and Iquitos, Peru; 4 Fogarty International Center, National Institutes of Health, Bethesda, Maryland, United States of America; 5 Department of Global Health Systems and Development, Tulane University School of Public Health and Tropical Medicine, New Orleans, Louisiana, United States of America; 6 Graduate School of Public Health, San Diego State University, San Diego, California, United States of America; Yale School of Public Health, United States of America

## Abstract

**Background:**

Empiric evidence shows that *Aedes aegypti* abundance is spatially heterogeneous and that some areas and larval habitats produce more mosquitoes than others. There is a knowledge gap, however, with regards to the temporal persistence of such *Ae. aegypti* abundance hotspots. In this study, we used a longitudinal entomologic dataset from the city of Iquitos, Peru, to (1) quantify the spatial clustering patterns of adult *Ae. aegypti* and pupae counts per house, (2) determine overlap between clusters, (3) quantify the temporal stability of clusters over nine entomologic surveys spaced four months apart, and (4) quantify the extent of clustering at the household and neighborhood levels.

**Methodologies/Principal Findings:**

Data from 13,662 household entomological visits performed in two Iquitos neighborhoods differing in *Ae. aegypti* abundance and dengue virus transmission was analyzed using global and local spatial statistics. The location and extent of *Ae. aegypti* pupae and adult hotspots (i.e., small groups of houses with significantly [p<0.05] high mosquito abundance) were calculated for each of the 9 entomologic surveys. The extent of clustering was used to quantify the probability of finding spatially correlated populations. Our analyses indicate that *Ae. aegypti* distribution was highly focal (most clusters do not extend beyond 30 meters) and that hotspots of high vector abundance were common on every survey date, but they were temporally unstable over the period of study.

**Conclusions/Significance:**

Our findings have implications for understanding *Ae. aegypti* distribution and for the design of surveillance and control activities relying on household-level data. In settings like Iquitos, where there is a relatively low percentage of *Ae. aegypti* in permanent water-holding containers, identifying and targeting key premises will be significantly challenged by shifting hotspots of *Ae. aegypti* infestation. Focusing efforts in large geographic areas with historically high levels of transmission may be more effective than targeting *Ae. aegypti* hotspots.

## Author Summary

We carried out a comprehensive study of the long-term trends in household-level *Aedes aegypti* spatial distribution within a well-defined urban area endemic for dengue virus. By using a dataset consisting of 13,662 household entomological visits performed in two neighborhoods in Iquitos, Peru, we quantified the ∼3 year spatial clustering patterns of *Ae. aegypti* among houses and the temporal persistence of vector abundance hotspots. Our results provide strong support for the conclusion that *Ae. aegypti* distribution is highly focal and that hotspots of high vector abundance at the level of small groups of houses are common, but temporally unstable. Results from our study have implications for understanding the spatio-temporal patterns of *Ae. aegypti* abundance and for the design of surveillance and control activities that are based on household-level entomological data.

## Introduction

Despite decades of vector control efforts, dengue has become the most important mosquito-borne viral disease of humans. Estimates indicate that ∼390 million dengue virus (DENV) infections occur annually throughout the tropical and subtropical world [1], [2]. In the last twenty years, dengue epidemics have increased in number, magnitude and severity, due in part to range expansion of the mosquito vector *Aedes aegypti*, geographic spread and evolution of DENV, and increased urbanization and international travel [3], [4]. The emergence of DENV as a public health problem has been influenced by the interplay of multiple factors, including the abundance, dispersal and blood feeding patterns of female *Ae. aegypti*; complex interactions among multiple virus serotypes and genotypes; environmental factors (i.e., temperature, humidity and rainfall); herd immunity in human populations; and human density, age structure and movement [4]–[8].

*Aedes aegypti's* ecology and behavior contribute to its efficient transmission of DENV and spatio-temporal patterns of human DENV infections. They bite during the daytime when human hosts are active, are highly anthropophilic, are well adapted to human habitations, and tend to be relatively sedentary with limited dispersal tendencies; they seldom disperse beyond 100 m [7], [9]–[12]. Results from mathematical and simulation models indicate that such traits can have strong effects on DENV transmission dynamics, due to their influence on contact between humans and mosquito vectors [13], [14]. Empiric evidence from entomologic field surveys and population genetics studies support the notion that *Ae. aegypti* abundance is spatially heterogeneous and that some areas and larval habitats are likely to produce more adult mosquitoes than others [15]–[21]. Given the current emphasis on spatially-based interventions (where reactive control is performed based on the proximity to residences of dengue cases, [22]), identifying and predicting the occurrence of vector hotspots (small groups of houses with disproportionately high productivity, vector abundance and potential for DENV transmission) is a logical next step for assessing current control recommendations and devising innovative concepts for *Ae. aegypti* control and dengue management.

Understanding patterns of *Ae. aegypti* distribution at fine spatial (e.g., at the household level and within a neighborhood) and temporal scales (e.g., within and across consecutive seasons and years), is curtailed by the difficulty of collecting adequate information at those levels of resolution. Most studies describing within-city patterns of *Ae. aegypti* distribution are performed at aggregated spatial scales (neighborhoods or census districts, e.g. [23], [24]) or by analyzing information from a network of traps spaced over several hundred meters (e.g. [19], [25]). In one detailed analysis, Getis et al. [15] reported that, in Iquitos, Peru adult *Ae. aegypti* were aggregated up to 30 meters, but pupae did not cluster beyond the household. Their findings are in agreement with the focal nature of *Ae. aegypti* dispersal [9] and have been validated in rural Thai villages [20], [26], [27], in a northern Argentina community neighborhood [28], in coastal Ecuador [29], and through complex simulation models [30], [31]. Implicit in the finding of spatially clustered populations is the notion that hotspots of high vector abundance could be the focus of targeted vector control interventions. Theoretical models support the idea that control interventions targeting hotspots can disproportionately reduce pathogen transmission in comparison to blanket or random interventions [32]–[38]. Furthermore, targeting vector control interventions with greater or equal efficacy to blanket interventions could also result in reduced pesticide usage and operational costs (e.g., [38], [39]).

Most of the published research on the spatial pattern of household-level *Ae. aegypti* distribution covered short temporal scales (either cross-sectional or a single season), analyzed data on vector presence but not abundance, lacked measures of the variability of clustering estimates, and did not consider how persistent (or predictable) spatial clusters were. Before considering whether *Ae. aegypti* hotspots could be considered as rational targets for vector control, information on their temporal variability and persistence is required to better inform where and when interventions should occur. To fill this knowledge gap, we used a detailed longitudinal entomologic dataset to quantify long-term patterns of *Ae. aegypti* spatial distribution at the household level. Specifically, our objectives were to (1) quantify the spatial clustering patterns of adult female *Ae. aegypti* and pupae counts per house over nine sampling surveys separated by approximately four months, spanning a 3-year period (2009–2011) in two Iquitos neighborhoods differing in *Ae. aegypti* infestation levels; (2) determine overlap between clusters of *Ae. aegypti* females and pupae; (3) quantify the spatial and temporal stability of clusters over the nine entomologic surveys.

## Materials and Methods

### Ethics statement

Strict protocols for household enrolment study were followed, including contacting homeowners and asking for their permission to have their house and patio being inspected for pupae and adult mosquito presence and abundance. The procedures for enrollment of households in the entomologic and demographic surveys were approved by University of California, Davis (2007.15244); NAMRU-6 (NMRCD 2007.0007), which included Peruvian representation; and Emory University (IRB9162) Institutional Review Boards.

### Study design and data collection

Our study was performed in the Maynas and Tupac Amaru neighborhoods of the city of Iquitos, the largest urban center (population ∼370,000) in the Peruvian Amazon. The two neighborhoods were described previously [24], [40], [41] and were chosen for comparison because they differed epidemiologically and entomologically. Maynas has higher DENV prevalence rates and *Ae. aegypti* infestation levels than Tupac Amaru [15]. Additionally, Maynas is older, more centrally located within Iquitos, more urbanized and wealthier than Tupac Amaru [24], [40], [41].

Data were collected using standardized household entomological surveys performed approximately every 4 months from 2009 to 2012 (9 consecutive surveys). Methods for surveys and mosquito collection are described in detail elsewhere [15], [24]. Briefly, *Ae. aegypti* productivity was assessed by pupal surveys performed for all containers at each surveyed house [24]. Indoor and outdoor adult mosquito abundances were measured by using Prokopack mosquito aspirators [42]. Two-person survey teams were rotated over time to limit temporal and collector bias [24]. Aspiration collections were conducted in each room of the house as well as in the patio. All containers found to be holding water were measured, classified, and scored for sunlight exposure, fill method (actively via faucet or passively by rain), and presence of a cover. Collected adults and pupae were taken to the field laboratory for species identification. Pupae were counted and placed in plastic vials labeled with a unique house number, container code, and date. Each subsequent day, adults that emerged were collected and placed in a −20°C freezer. After 30 minutes to 1 hour, they were identified to species, counted by sex, and data were recorded on the entomology collection sheet.

All collected data were linked to a household level Geographic Information System (GIS) for the city of Iquitos (described in [15], [24], [43]). Survey data was imported into ArcGIS 10.1 (ESRI, Redlands, CA) and linked to the Iquitos GIS by the house code (a unique alpha-numeric code painted on every house's door and used throughout the Iquitos field studies). Data were then projected in Universal Transverse Mercator and WGS −84 DATUM and used to map the raw field data as well as results of spatial statistics tests.

### Statistical analyses

Spatial analyses were performed on the number of water-holding containers per house (a measure of habitat availability), the number of *Ae. aegypti* pupae per house (a measure of productivity) and number of *Ae. aegypti* adult males and females per house (a measure of DENV entomologic risk). Details of each test formula, expected values, and calculations were thoroughly described by Getis et al. [15]. Below, we provide a brief description of each statistical test and its implementation within the context of this study.

Global spatial statistics were implemented to detect the presence of spatial clustering of *Ae. aegypti* infestation anywhere within each study neighborhood [15]. To account for bias introduced by the clustered pattern of households within a block we compared the increments in the observed clustering of houses (k-function) with the pattern and of *Ae. aegypti* presence (k-function) and abundance (weighted k-function) as described by Getis et al. [15]. A bivariate k-function test [44] was implemented to detect the spatial scale up to which pupae and adult presence were related to each other. The function was an extension of the k-function for univariate data and compared the scales up to which infestation in one event (e.g., pupae) were more clustered than the distribution of the two events combined (infestation of pupae and adults). Given that points located on the edges were more likely to cluster because they had fewer neighbors than central points, an edge effect correction was included in the formulae of all k-functions [15].

Local Getis hotspot analysis (G_i_*) was applied to map the occurrence of clusters of high *Ae. aegypti* abundance and water-holding container numbers [15]. Houses that were members of clusters were identified using a z-score of ±3.706 as a cutoff for cluster membership (Bonferroni-corrected z-value). To account for overdispersion in the data (which can dramatically affect Getis G_i_* test), analyses were performed on the log-transformed mosquito abundance (Log[number of pupae/adults+1]) and the log-transformed number of water-holding containers (Log[number of containers+1]). Once members of significant clusters were identified, the distance up to which clustering occurred around each house was identified as by Getis et al 2003 [15]. Local analyses were performed separately for each neighborhood, entomologic survey and *Ae. aegypti* infestation measure. For all spatial analysis tests, clustering distances between 1–5 meters were considered to occur within the household (the average width of a house lot in Iquitos is 4.6 m) whereas clustering distances beyond 5 m were considered to be between households. Weighted K-function analysis was performed on the aggregated number of entomologic surveys that a house was member of a high *Ae. aegypti* abundance cluster (range of values, 1–9 surveys) to determine whether some houses or areas within each neighborhood were consistent hotspots of vector abundance. In the context of our study, we define an *Ae. aegypti* hotspot as a distinct house or group of houses with significantly higher mosquito densities than surrounding houses [33], [36].

Clustering distances of adult *Ae aegypti* at the household level (from the Getis G_i_* test) aggregated across all entomologic surveys were used to calculate the cumulative probability distribution of clustering; i.e., the probability of finding clusters with an extent equal to or less than *d* meters. Maximum Likelihood techniques were applied to fit various statistical distributions (e.g., exponential, power law) to the cumulative probability distribution of the distance of local clustering. This kind of functional relationship described the probability of finding spatially correlated populations at increasing distances from a household. Curve fits were performed independently for each neighborhood and for both neighborhoods combined.

Analyses were performed using the Point Pattern Analysis (PPA) program developed by Arthur Getis with assistance from Laura Hungerford, Dong-Mei Chen, and Jared Aldstadt (available online at http://www.nku.edu/~longa/cgi-bin/cgi-tcl-examples/generic/ppa/ppa.cgi) and the packages splancs ([45]) and fitdistrplus ([46]) of the R statistical software (ver 2.15 [47]). Curve fitting procedures were performed using Matlab (Mathworks, Natick, MA) curve fitting function.

## Results

### Neighborhood characteristics

From March 2009 to October 2011, 13,662 household entomological inspections were performed (7,156 in Maynas and 6,506 in Tupac Amaru; Table 1). A total of 1,226 and 1,068 unique houses were inspected in Maynas and Tupac Amaru, respectively (Table 1). On average (SD), 884 (59) and 832 (73) households were visited on each entomologic survey in Maynas and Tupac Amaru, respectively. Seventy-seven percent (SD = 7.5%) of buildings surveyed in both neighborhoods were residential, followed by stores (mainly houses used as neighborhood stores) (mean = 17.5%; SD = 1.8%) (Figure S1). Surveys lasted on average (SD) 22 (7) days in Maynas and 18 (5) days in Tupac Amaru (Table 1). On average (SD), each house was surveyed 5.8 (2.4) times throughout the study period. The average (SD) number of residents per house in both neighborhoods was 6.0 (3.1). Percentage of households using two of the most important water sources (piped and rain water) ranged from 91% to 95% for piped and 2% to 7% for rain water.

**Table 1:** Description of entomologic surveys and aggregate measures of *Aedes aegypti* infestation within the Maynas and Tupac Amaru neighborhoods of Iquitos, Peru.

		Survey Dates			Adults		
Neighborhood	Survey No.	Start	End	Houses Surveyed (N)	All Adults (N)	Adult Females (N)	Pupae (N)
**Maynas**	**1**	3/19/09	4/3/09	781	162	106	642
	**2**	7/14/09	8/6/09	837	318	142	579
	**3**	11/30/09	12/14/09	692	691	327	253
	**4**	3/24/10	4/29/10	835	870	383	381
	**5**	8/18/10	9/13/10	744	1289	631	272
	**6**	12/27/10	1/18/11	772	1016	535	221
	**7**	4/28/11	5/17/11	809	322	155	346
	**8**	8/16/11	9/12/11	884	644	301	498
	**9**	1/13/12	2/1/12	802	359	165	
**Tupac Amaru**	**1**	3/4/09	3/18/09	706	49	31	298
	**2**	6/26/09	7/13/09	694	173	104	662
	**3**	11/13/09	11/27/09	637	375	204	211
	**4**	2/25/10	3/11/10	692	351	164	219
	**5**	7/2/10	7/20/10	670	338	161	354
	**6**	10/6/10	10/27/10	671	612	330	279
	**7**	2/23/11	3/22/11	832	255	115	106
	**8**	7/4/11	7/20/11	802	581	269	110
	**9**	10/24/11	11/15/11	802	304	174	402

**Figure 1:**
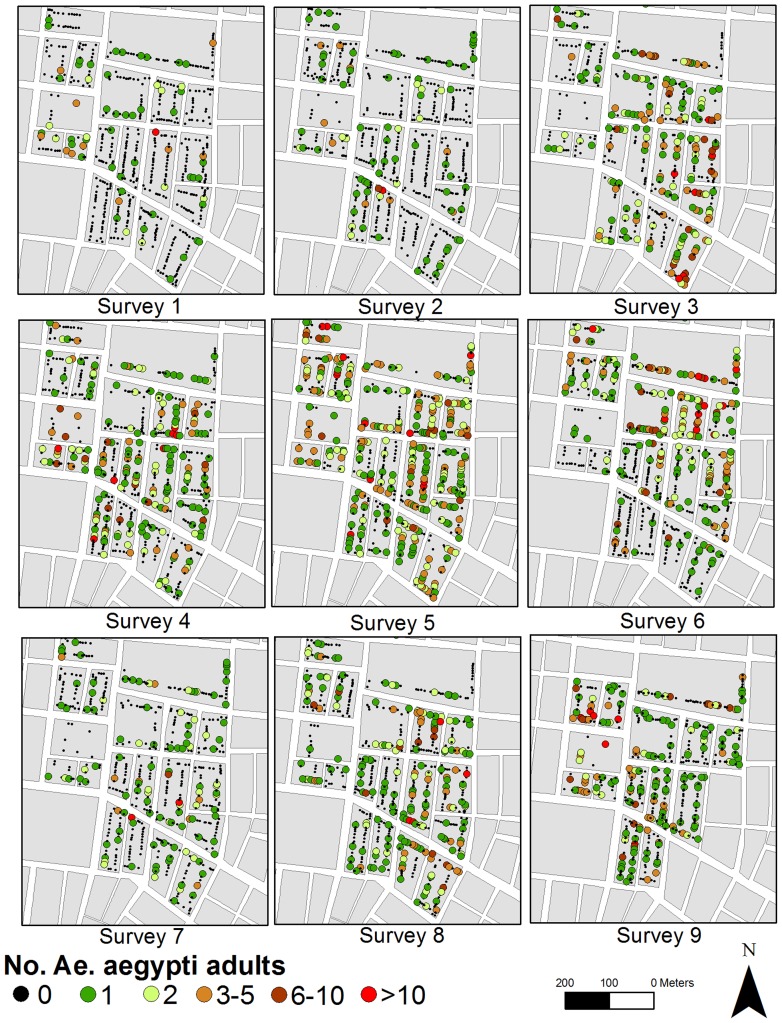
Numbers of adult male and female *Ae. aegypti* collected per house and entomologic survey in Maynas neighborhood, Iquitos, Peru. Refer to Table 1 for information about each entomologic survey.

**Figure 2:**
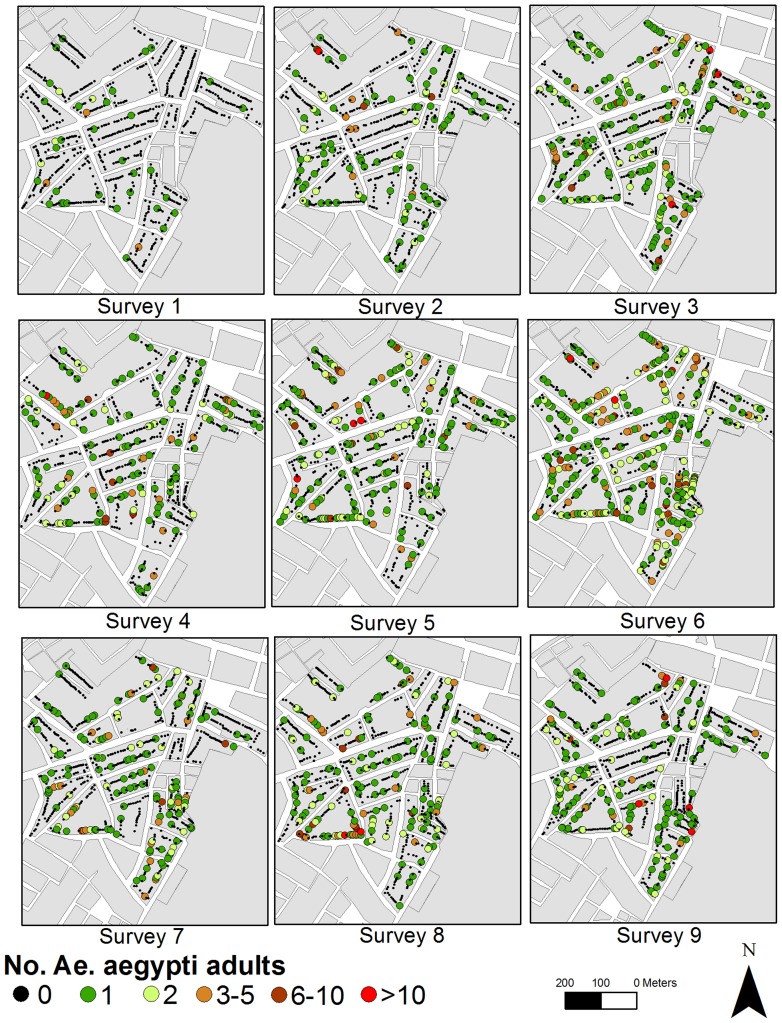
Numbers of adult male and female *Ae. aegypti* collected per house and entomologic survey in Tupac Amaru neighborhood, Iquitos, Peru. Refer to Table 1 for information about each entomologic survey.

Maynas households had a significantly higher average number of water-holding containers than Tupac Amaru (Two-sample Wilcoxon test, W = 224207, P<0.001). Across both neighborhoods, most (98.9%) houses had at least one water holding container throughout the study period. The proportion of houses with positive containers ranged between 0.04–0.12 in Maynas and 0.03–0.10 in Tupac Amaru (Figure S2). A total of 5,833 *Ae. aegypti* pupae (3,192 in Maynas and 2,641 in Tupac Amaru) and 8,709 adult males and females (5,671 in Maynas and 3,038 in Tupac Amaru) were collected over the nine surveys. Forty-nine percent of all *Ae. aegypti* adults collected were females. Adult abundance was highly overdispersed, 91% of all Maynas houses and 94.9% of all Tupac Amaru houses were infested with 5 or less adult *Ae. aegypti* mosquitoes. The median number of adult *Ae. aegypti* per house was significantly higher in Maynas than in Tupac Amaru (Figure S3, W = 1544194, P<0.001). There was, however, considerable variation embedded in these estimates (Figure 1 and Figure 2). The number of adults and pupae collected per house ranged from 0 to 163 and from 0 to 681, respectively. Infested houses were found throughout the study neighborhoods (Figure 1 and Figure 2).

### Global spatial patterns of pupae and adult infestation

Table 2 summarizes the results of the k-functions applied to pupae and adult presence and abundance in each neighborhood. Pupal collections showed a low degree of spatial clustering in both neighborhoods (Table 2). Clusters of pupae presence and abundance were observed in 44% and 11% of surveys, respectively, for Maynas and in 55% and 33% of surveys in Tupac Amaru (Table 2). The estimated overall mean ± SD clustering distance was 16.6±5.0 m for pupae presence and 10.3±7.8 m for pupae abundance. Average clustering distances did not differ between neighborhoods (17.5±5.5 m in Maynas vs 16.0±5.0 m in Tupac Amaru). The spatial distribution of adult *Ae. aegypti* showed a stronger pattern, with clusters found on every survey. Clustering distances in both neighborhoods ranged from 1 m (within the household) to 40 m, with mean ± SD clustering values across both neighborhoods for adult presence and abundance of 16.3±10.7 m and 17.4±12.9 m, respectively (Table 2).

**Table 2:** Result of global clustering tests applied to the presence of pupae and adult *Ae. aegypti* (k-function) and to the abundance of pupae and adult *Ae. aegypti* (weighted k-function) in the Maynas and Tupac Amaru neighborhoods of Iquitos, Peru.

		Spatial clustering distance (meters)			
		Pupae		Adult males & females	
Neighborhood	Survey	Presence	Abundance	Presence	Abundance
**Maynas**	**1**	NS*	NS	1**	1
	**2**	NS	NS	20	20
	**3**	10	NS	20	10
	**4**	20	NS	10	20
	**5**	NS	NS	20	30
	**6**	20	20	20	10
	**7**	NS	NS	1	1
	**8**	NS	NS	40	40
	**9**	20	NS	20	30
	Average (SD)	17.5 (5.0)	20	17.8 (10.6)	18.9 (12.4)
**Tupac Amaru**	**1**	NS	NS	1	1
	**2**	10	NS	10	10
	**3**	10	1	10	20
	**4**	20	NS	30	20
	**5**	NS	10	20	30
	**6**	20	10	10	1
	**7**	NS	NS	20	10
	**8**	20	NS	30	40
	**9**	NS	NS	10	20
	Average (SD)	16.0 (5.5)	8.3 (2.9)	16.1 (9.3)	17.8 (11.8)
**Overall average (SD)**		16.6 (5.0)	10.3 (7.8)	16.3 (10.7)	17.4 (12.9)

*NS indicates that the observed pattern was not statistically significantly different than random.

**A distance below 5 meters means that clustering occurred only at the household where collections were performed (average house width in Iquitos is ∼4.6 m).

**Figure 3:**
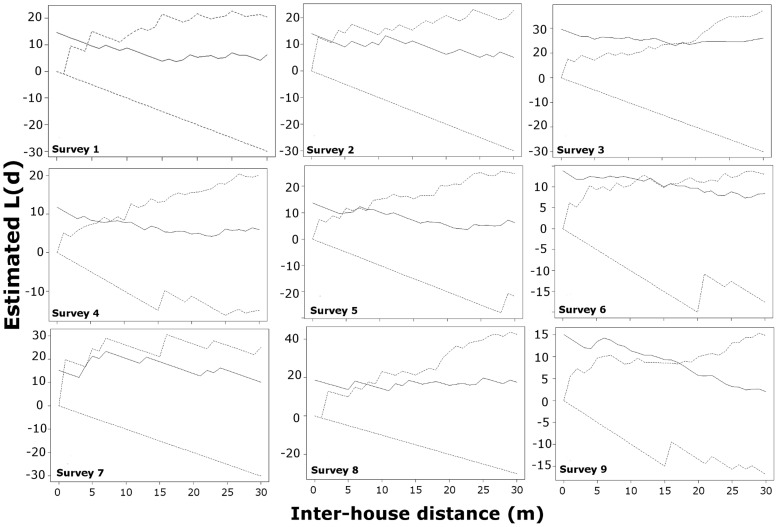
Spatial correlation between pupae and adult presence. Results from the bivariate k-function quantifying the scales of spatial association between pupae and adult male and female *Ae. aegypti* presence during 9 entomologic surveys spanning between 2009 and 2011. Number inside each plot indicates the survey number. Solid lines represent observed values whereas dashed lines the random expectation for an alpha value of 0.05. Analyses were performed to the combined dataset of the Maynas and Tupac Amaru neighborhoods.

Figure 3 shows the results of the bivariate k-functions applied to test the scale up to which pupae and adult infestation were associated during nine entomologic surveys between 2009 and 2011. Given the lack of difference in global clustering between neighborhoods, results were pooled to show the overall scale up to which pupae and adults are associated. When the observed value (solid line) is higher than the random expectation (dashed line) spatial association between variables occurs at such a distance (Figure 3). For 4 out of 9 surveys (44%), pupae and adults were clustered within the household (at a distance of 5 m or less) (Figure 3). A very focal level of association between pupae and adults was found when clustering occurred beyond the household (Figure 3); the average ±SD clustering distance was 11.4±5.4 m. Collections performed in December-January (surveys 3, 6 and 9) had higher extent of association between pupae and adults (15–17 meters) compared to the remaining surveys (up to 5 m), indicating that during those months either the extent of populations is larger or the abundance of *Ae. aegypti* is more patchily distributed.

### Local spatial patterns of adult infestation and container availability

The number of water-holding containers (log+1)-transformed did not show any strong spatial pattern. Out of an average of 722 houses per survey in Tupac Amaru, only 5 unique households were members of significant clusters (GI*(d)>3.71; P<0.05) of high container numbers (one in survey 2, two in survey 4, one in survey 5 and one in survey 6). In Maynas, only three houses were members of clusters (all in survey 6). Clustering distances in all cases did not exceed the household (<5 m). This indicates that, whereas water-holding containers are very common, they do not show any spatial structure within both neighborhoods.

Given the low probability of finding pupae clusters, local spatial analyses were performed on *Ae. aegypti* adult abundance data only. Hotspot analysis maps are presented in Figure S3 and summaries of clustering measures in Table 3. On average, 3.1% of Maynas and 1% of Tupac Amaru households were members of a cluster of high adult abundance (Table 3 and Figure S3). An average of 51.8% (range = 32–68%) of all adults collected in Maynas and 28.7% (10–54%) of all adults collected in Tupac Amaru were found within the identified spatial clusters. In Maynas, an average of 30.4% (range = 0–53%) of clusters of adult abundance occurred beyond the household, whereas in Tupac Amaru the proportion of clusters occurring beyond the household increased to 50.7% (range = 14–86%) (Table 3).

**Table 3:** Summary results from local hot-spot analyses performed on the household numbers of adult males and female *Ae. aegypti* (Log-transformed) per entomologic survey in Maynas and Tupac Amaru neighborhoods.

	Proportion of surveyed houses that are hot-spots		Proportion of total *Ae. aegypti* within a hot-spot		Proportion of hot-spots beyond the household	
Survey	Maynas	Tupac Amaru	Maynas	Tupac Amaru	Maynas	Tupac Amaru
1	0.024	0.01	0.60	0.35	0.40	0.57
2	0.024	0.01	0.65	0.30	0.00	0.57
3	0.031	0.01	0.36	0.21	0.33	0.14
4	0.042	0.01	0.60	0.38	0.28	0.57
5	0.027	0.01	0.32	0.20	0.18	0.57
6	0.034	0.01	0.56	0.10	0.38	0.28
7	0.017	0.01	0.46	0.18	0.14	0.57
8	0.037	0.01	0.68	0.54	0.53	0.43
9	0.045	0.01	0.43	0.32	0.50	0.86
Mean (SD)	0.03 (0.009)	0.01 (0)	0.52 (0.13)	0.29 (0.13)	0.30 (0.17)	0.51 (0.21)

**Figure 4:**
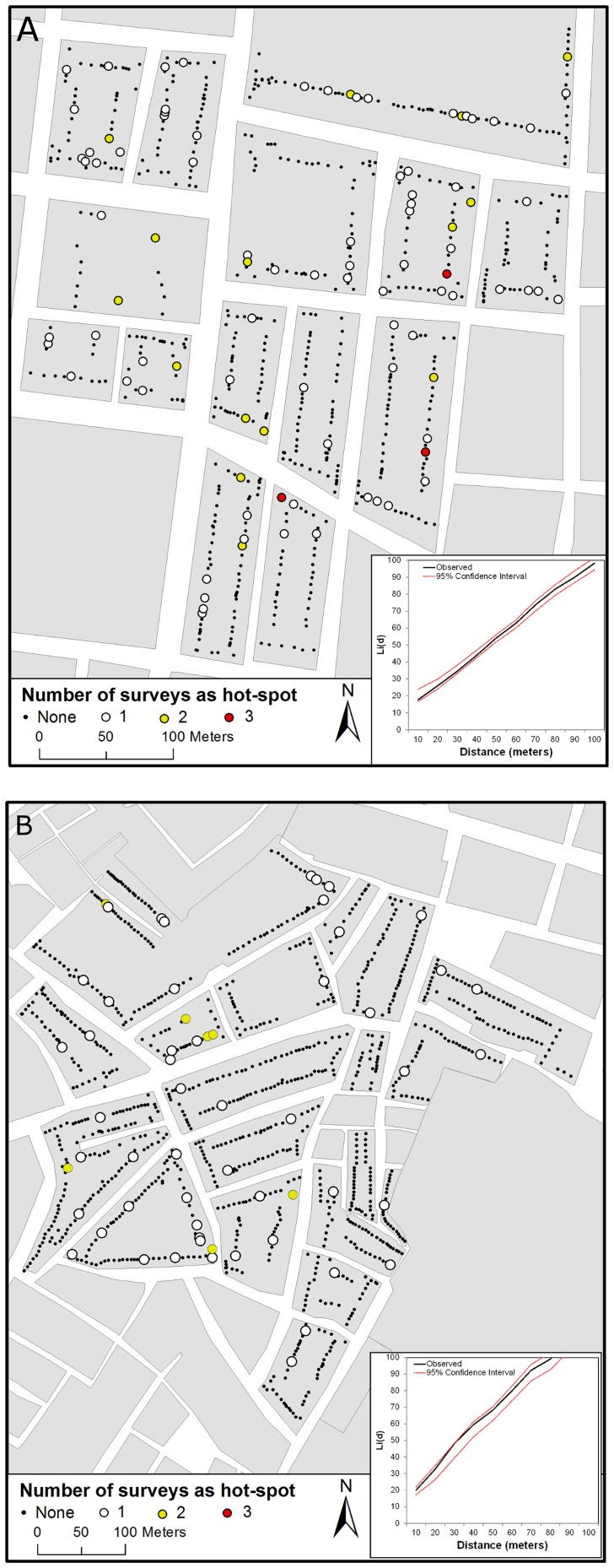
Temporal instability in Ae. aegypti clusters. Maps show the number of surveys (out of 9 total surveys) a house was a hot-spot of high adult male and female *Ae. aegypti* abundance for (A) Maynas and (B) Tupac Amaru neighborhoods. Inset in each panel show the result of weighted k-function analysis performed on the number of times a house was a hot-spot. Global clustering occurs when observed values (solid black line) are higher than the expected 95% CI under a random distribution (red dotted lines).

There was no obvious consistent temporal pattern of adult clusters in both neighborhoods; i.e., the location of clusters in one survey differed from the location of clusters in future or prior surveys. The temporal instability in *Ae. aegypti* hotspots is shown in Figure 4. Most houses in Maynas and Tupac Amaru (80.9% and 87.9%, respectively) were identified as hotspots only once in the 9 survey periods. The maximum number of survey dates when a house was identified as a hotspot was 3 (out of 9 surveys) in Maynas and 2 (out of 9 surveys) in Tupac Amaru (Figure 4). The spatial location of hotspots did not follow any apparent spatial pattern; the distribution of hotspots within both neighborhoods did not differ from a random distribution (Figure 4).

**Figure 5:**
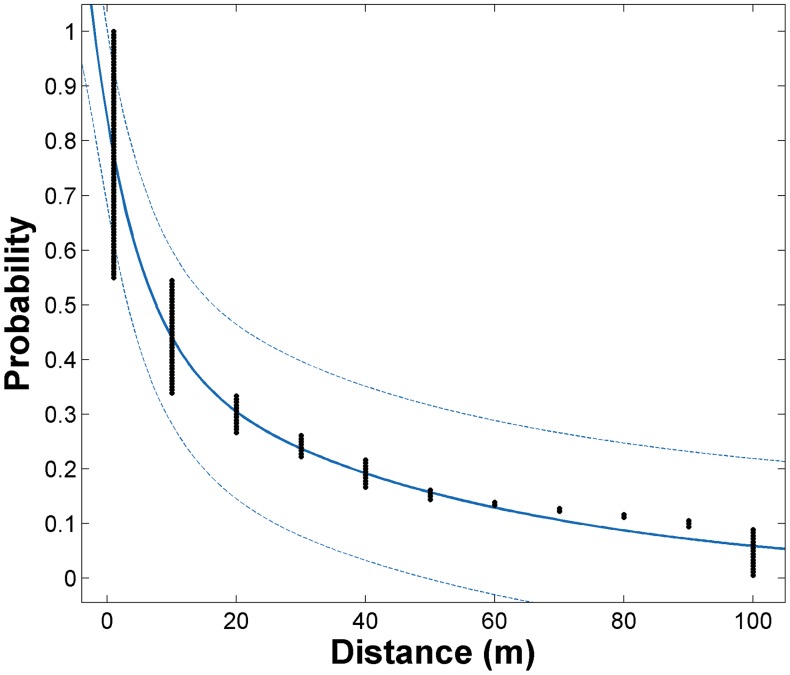
Probability of finding spatially correlated adult male and female *Ae. aegypti* populations at increasing distances from a household. Points integrate data from 9 entomologic surveys performed in the Maynas and Tupac Amaru neighborhoods during 2009–2011 and solid line shows exponential fit results together with its 95% confidence interval (dotted line). Refer to Table 4 for model fit results.

We used the maximum distance of adult *Ae aegypti* local clustering in each neighborhood to estimate the cumulative probability distribution for finding spatially correlated populations at increasing distances from a household (Figure 5). A value of 0–5 meters in the X-axis of Figure 5 indicates that clustering did not exceed the household whereas values higher than 5 meters indicate that *Ae. aegypti* abundance was spatially correlated beyond the household. The probability of finding spatially correlated adult populations decreased significantly with increasing distances from the house, with patterns for both neighborhoods better explained by a negative exponential model of the form 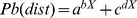 (Table 4). Model fit was very high (R^2^_Maynas_ = 0.91; R^2^_Tupac Amaru_ = 0.86; R^2^_Both_ = 0.89). When data from both neighborhoods was combined, the probability of finding adults clustering beyond the household (>5 m) was 42% (95% CI,57.8–25.8%) and the finding of clusters of high adult abundance with an extent of 100 m was rare (6.1%, 95% CI, 0.0–21.0%) (Figure 5 and Table 4). Predicted values were very similar between neighborhoods (Table 4 and Figure S5).

**Table 4:** Model fit parameters to the cumulative probability distribution of the distance up to which clustering of adult male and female *Ae. aegypti* populations occurred.

	Estimate (95% CI)		
Parameter	Maynas	Tupac Amaru	Both
a	0.415 (0.237, 0.592)	0.398 (−0.170, 0.967)	0.425 (0.243, 0.606)
b	−0.150 (−0.270, −0.030)	−0.156 (−0.475, 0.162)	−0.146 (−0.250, −0.042)
c	0.442 (0.246, 0.639)	0.424 (−0.194, 1.04)	0.416 (0.219, 0.614)
d	−0.017 (−0.026, −0.009)	−0.027 (−0.06, 0.011)	−0.020 (−0.030, −0.009)
**Model Fit**			
R^2^	0.9079	0.8617	0.8942
RMSE*	0.08917	0.1108	0.09494

*Root Mean square error. Observed data was better explained by a function of the form 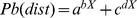 (see Figure 5 for plot of fitted model).

## Discussion

Trends over a 3-year study period in household-level spatial distribution within a well-defined urban area provide strong evidence for highly focal distribution of *Ae. aegypti*. Hotspots of high mosquito abundance in small groups of houses were common, but temporally unstable.

Theory predicts that interventions targeting super-spreaders can disproportionately impact pathogen transmission in comparison to blanket or non-targeted interventions [32], [34], [35]. For certain vector-borne diseases, locations are more important than individual persons with regard to their contribution to transmission; disease ‘hotspots’ or ‘key-locations’ dominate the spatial dynamics of various vector-borne diseases [33], [36], [38], [48]. For dengue, the concept of key locations has been studied in terms of productivity of larval habitats, leading to the identification of key-premises [49] and, via spatial analyses, identifying potential vector or virus hotspots. Some researchers have concluded that targeting vector control at hotspots of high *Ae. aegypti* productivity will be a more effective and efficient use of available resources than traditional, more evenly applied interventions [20], [28], [29], [50].

The occurrence of shifting hotspots of *Ae. aegypti* abundance imposes a significant challenge to intervention strategies targeting vector control on households. Because adult mosquito hotspots observed during one of our surveys did not predict hotspots at the same location during prior or subsequent surveys, we do not expect identifying and targeting key-premises [49] to be operationally practical in all DENV endemic settings. In most DENV endemic areas, the availability and type of containers that can produce adult *Ae. aegypti* are affected by the reliability of piped water services, a factor that tends to be highly variable in space and time [51]. Container management practices by the occupants of the property, coupled with the range of *Ae. aegypti* flight dispersal and *Ae. aegypti* egg-laying behavior further contribute to the spatially and temporally heterogeneous pattern of vector larval productivity and adult distribution [9], [18], [20], [50], [52]. Thus, in a city like Iquitos, where there is a relatively low percentage of *Ae. aegypti* in permanent water holding containers [52], and where container availability is high across households, a strategy of identifying and targeting key premises will be significantly challenged by shifting hotspots of *Ae. aegypti* infestation.

Using a grid of 19 BG-Sentinel traps uniformly distributed at ∼130 m intervals and surveyed every 3 weeks, Barrera [19] described the distribution of adult *Ae. aegypti* abundance as temporally stable, with some traps consistently being members of clusters of high mosquito abundance. Similarly, analysis of weekly sentinel ovitrap data aggregated at the block or neighborhood levels indicated high levels of persistence in *Ae. aegypti* infestation patterns [23], [25]. Such patterns differ dramatically from the observed lack of persistence in infestation clustering reported when analyzing household-level *Ae aegypti* abundance data; i.e., this study and Garelli et al. [28]. Both studies indicate that, although hotspots of *Ae. aegypti* abundance are common, their specific location within a study neighborhood is different in every entomologic survey. Thus, whereas *Ae. aegypti* abundance appears to be spatially autocorrelated within weeks and at aggregated geographic units, over longer time scales (months) and at fine spatial resolution (household) the occurrence of shifting rather than temporally stable hotspots appears to be a common feature of vector distribution. By integrating our results with the ones found at aggregated spatial units (neighborhoods or census districts) we postulate that focusing efforts in large geographic areas with historically high levels of transmission within a city may be more effective than targeting households statistically identified as *Ae. aegypti* hotspots.

By following the same sampling and statistical methodologies, and by using information from roughly the same households as the ones studied by Getis et al. [15], we are able to confirm that *Ae. aegypti* adult distribution is highly focal, with average clustering not exceeding the household and its immediate neighbors. Also similar to Getis et al. [15], our study shows that clusters of high pupal abundance were rare and, when present, they rarely exceeded beyond a single household. These findings are in agreement with reports from Thailand indicating average local clustering values of 15 m [26] and from Ecuador with clustering values for pupae and adults of up to 20 m and 10 m, respectively [29], but differ from a recent report from Argentina reporting clusters of pupal abundance extending up to 400 m [28]. One of the main factors explaining the difference between studies relates to the methodology used to assess clustering. For example, Garelli et al. [28] analyzed data using a test that does not account for the inherent clustered pattern of houses within blocks. In our study, like Getis et al. [15], we accounted for such bias by comparing the distribution pattern of mosquitoes to the background distribution of households. The focal nature of *Ae. aegypti* distribution imposes important challenges to the integration of household-level information into predictive models of city-wide dynamics of vector distribution. An unresolved issue concerns tradeoffs in the cost and predictability of different strategies for assessing and responding to city-wide entomologic risk for DENV infection. For example, would it be more appropriate to implement (both in isolation and combined) quick, imperfect and spatially widespread entomologic indexes such as ovitraps or would it be better to use time consuming, more precise and spatially constrained indices, such as detailed adult/pupal indices?

Counting the absolute number of pupae in each larval development site has been recommended as a method for prioritizing containers requiring treatment in targeted larval development-site reduction strategies [52], [53]. Pupal counts are also considered a representative approximation of local adult mosquito populations [24], [53], and the pupae per person index is a frequently cited indicator for calculating a minimum threshold of pupal infestation for DENV transmission risk. Our study extends previous assessments of the association between pupae and adult abundance by showing that both indices rarely correlate with each other at spatial scales beyond the household and, when they do, they do so within 15 m of a house. Overall, the lack of proper consideration of spatial and temporal scales at which entomological measures are valid, as well as the limited inclusion of environmental, biological and human behavioral drivers of human-mosquito contacts, are important knowledge gaps in our ability to derive the maximum benefit out of entomological measures for surveillance and control programs [22].

The spatial pattern of *Ae. aegypti* distribution we detected was consistent across two neighborhoods that differed in mosquito infestation levels and DENV transmission. Maynas had high *Ae. aegypti* abundance and DENV transmission levels. Tupac Amaru had lower vector abundance and one of the lowest sero-incidence levels in the city of Iquitos [40], [41]. In both neighborhoods, however, *Ae. aegypti* populations were spatially clustered, clustering occurred at similar distances, and hotspots had a weak temporal persistence. Because most spatial analysis tests focus on relative rather than absolute patterns (i.e., compare observed values at location *i* to the overall mean), the finding of similar patterns in both neighborhoods may point to similar mechanisms driving *Ae. aegypti* population dynamics in them.

*Ae. aegypti* control is generally reactive (applied after the detection of local human DENV infections) and tends to rely on a geographic-based design in which interventions are applied at a given distance from a dengue case's residence [22]. Most programs use 100 m [54] as operational thresholds to deliver insecticides or other interventions, based on the premise that this distance represents the upper limit for *Ae. aegypti* dispersal. What this distance threshold does not take into account is that infected people can quickly move the virus well beyond 100 m of their home [6], [55], [56]. The lack of an empirically derived dispersal kernel (the probability of a given mosquito dispersing *d* meters away) for *Ae. aegypti* has further encouraged adoption of 100 m as the threshold for control measures. The focal pattern of *Ae. aegypti* adult distribution at the household level derived from our study suggests that adult flight beyond 30 m would be a rare event, provided food and habitat are available within such a radius. By integrating information on the extent of clustering of adult *Ae. aegypti* in two neighborhoods and over 9 entomologic surveys, we estimated the probability of finding spatially correlated populations, which could emerge due to dispersal and mixing of adult populations located in neighboring premises. We consider such estimates as a proxy of a dispersal kernel for adult *Ae. aegypti*. Our analysis indicates that, regardless of the background infestation levels in a neighborhood, the probability of finding *Ae. aegypti* adults dispersing beyond the house decreases exponentially with distance, being very low (∼6%) at 100 m. Our observations are in agreement with mark-release-recapture data suggesting that most individual adult *Ae. aegypti* do not fly far from the household where they developed as larvae (or were released as adults) [9], [57]–[59]. In addition to not accounting for longer range movements by virus infected humans (6), our results indicate that vector control activities applied at 100 m from a case's house will be a highly inefficient use of resources because it dramatically overestimates the actual extent of entomological risk associated with a potential transmission hotspot.

## Supporting Information

Figure S1 Relative distribution of all land-use types surveyed for adult and immature *Aedes aegypti* in the Maynas and Tupac Amaru neighborhoods of Iquitos, Peru. Refer to Table 1 for descriptions of each entomologic survey.
(JPG)

Figure S2 Proportion of surveyed houses with *Ae. aegypti* positive containers in Maynas and Tupac Amaru neighborhoods of Iquitos, Peru.
(JPG)

Figure S3 Median number (red line) and interquartile range of the number of adult male and female *Ae. aegypti* collected per house across nine entomologic surveys performed in the Maynas (MY) and Tupac Amaru (TA) neighborhoods of Iquitos, Peru.
(JPG)

Figure S4 Distance up to which adult male and female *Ae. aegypti* abundance clustered. Maps show the results of the Gi* tests by entomologic survey for Maynas (A) and Tupac Amaru (B) neighborhoods. Households for which no clusters were detected were labeled as NS (not significant).
(PDF)

Figure S5 Probability of finding spatially correlated adult male or female *Ae. aegypti* populations at increasing distances from a household. Panels show data from 9 entomologic surveys performed in (A) Maynas, (B) Tupac Amaru and (C) both neighborhoods combined. Solid line shows exponential fit results together with its 95% confidence interval (dotted line).
(PDF)
