# Supplementary figures and images for: Shifting Patterns of Aedes aegypti Fine Scale Spatial Clustering in Iquitos, Peru
Source: PLoS Negl Trop Dis. 2014 Aug 7;8(8):e3038. doi: 10.1371/journal.pntd.0003038 (PMC4125221; doi:10.1371/journal.pntd.0003038)

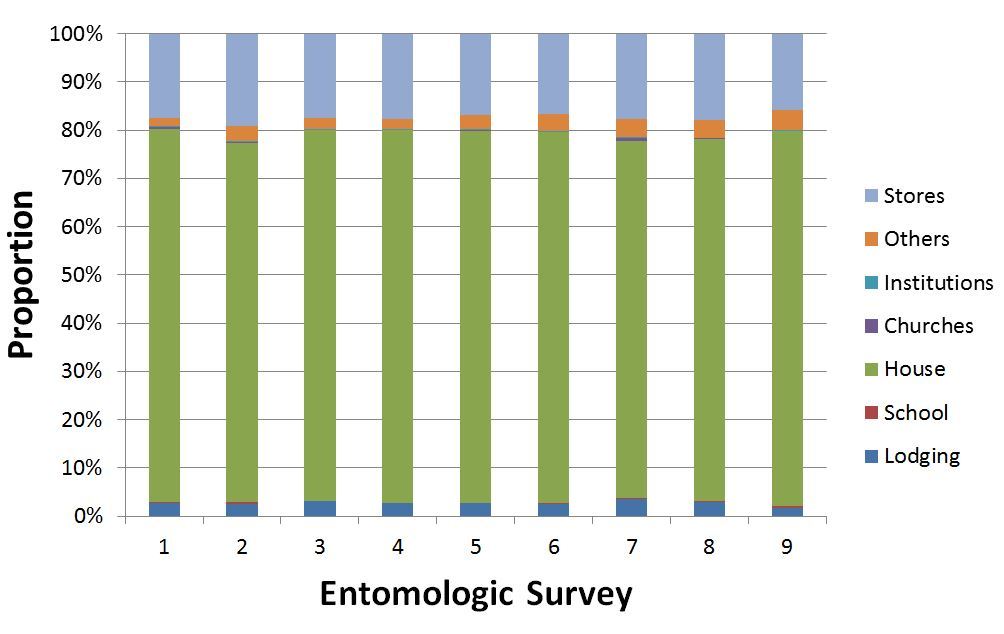

Supplement: Figure S1 — Relative distribution of all land-use types surveyed for adult and immature Aedes aegypti in the Maynas and Tupac Amaru neighborhoods of Iquitos, Peru. Refer to Table 1 for descriptions of each entomologic survey. (JPG) [file pntd.0003038.s001.jpg]

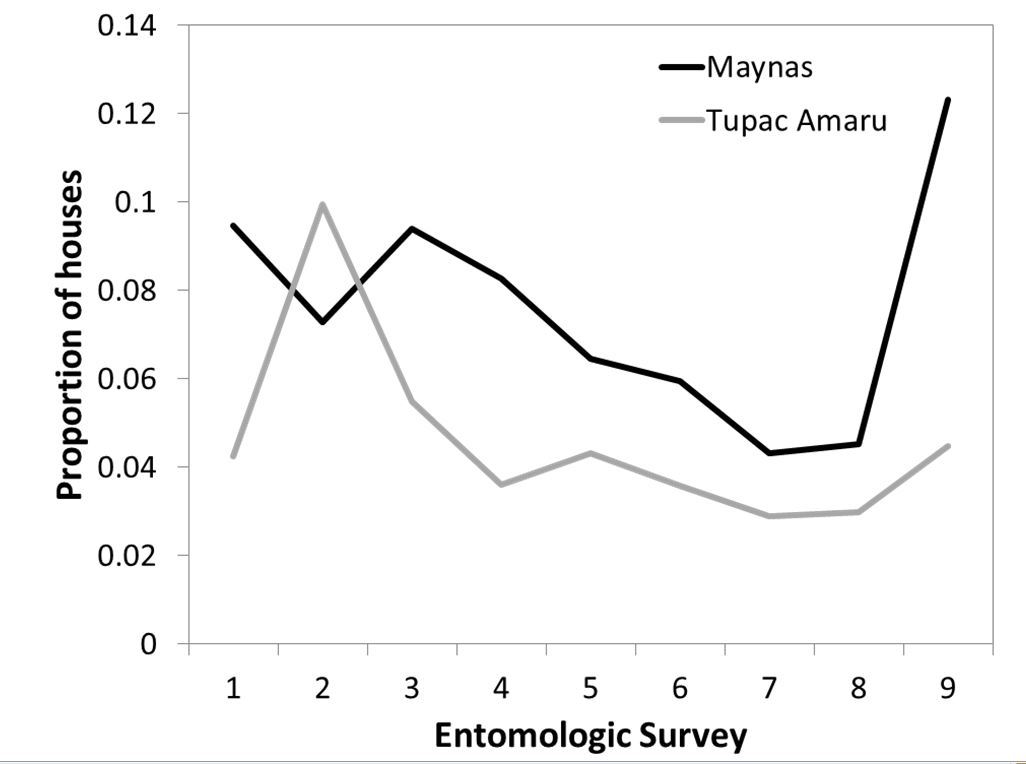

Supplement: Figure S2 — Proportion of surveyed houses with Ae. aegypti positive containers in Maynas and Tupac Amaru neighborhoods of Iquitos, Peru. (JPG) [file pntd.0003038.s002.jpg]

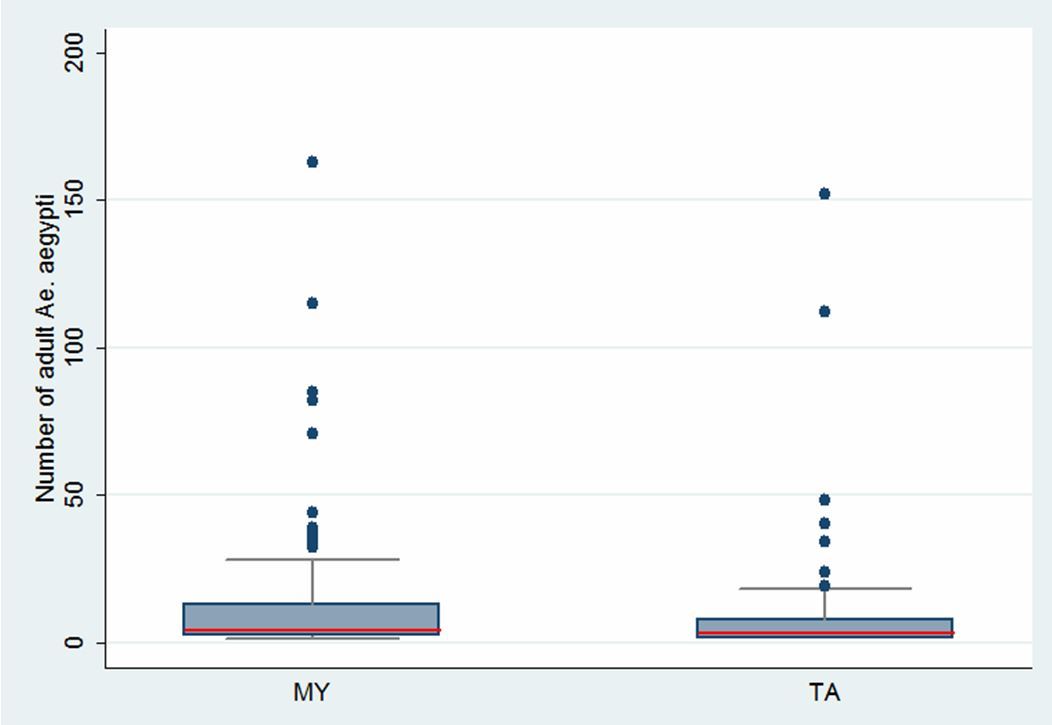

Supplement: Figure S3 — Median number (red line) and interquartile range of the number of adult male and female Ae. aegypti collected per house across nine entomologic surveys performed in the Maynas (MY) and Tupac Amaru (TA) neighborhoods of Iquitos, Peru. (JPG) [file pntd.0003038.s003.jpg]

(A)

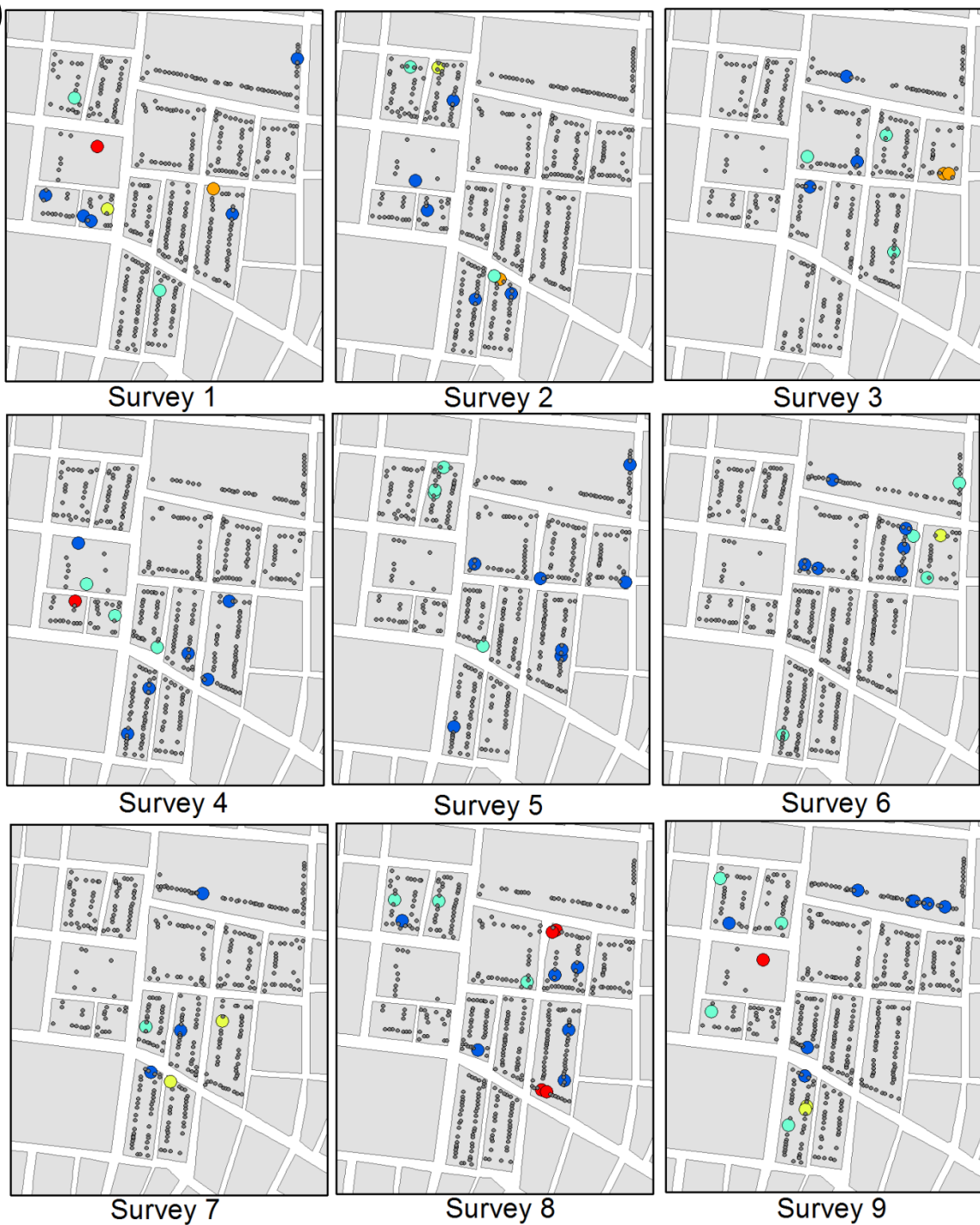

(B)

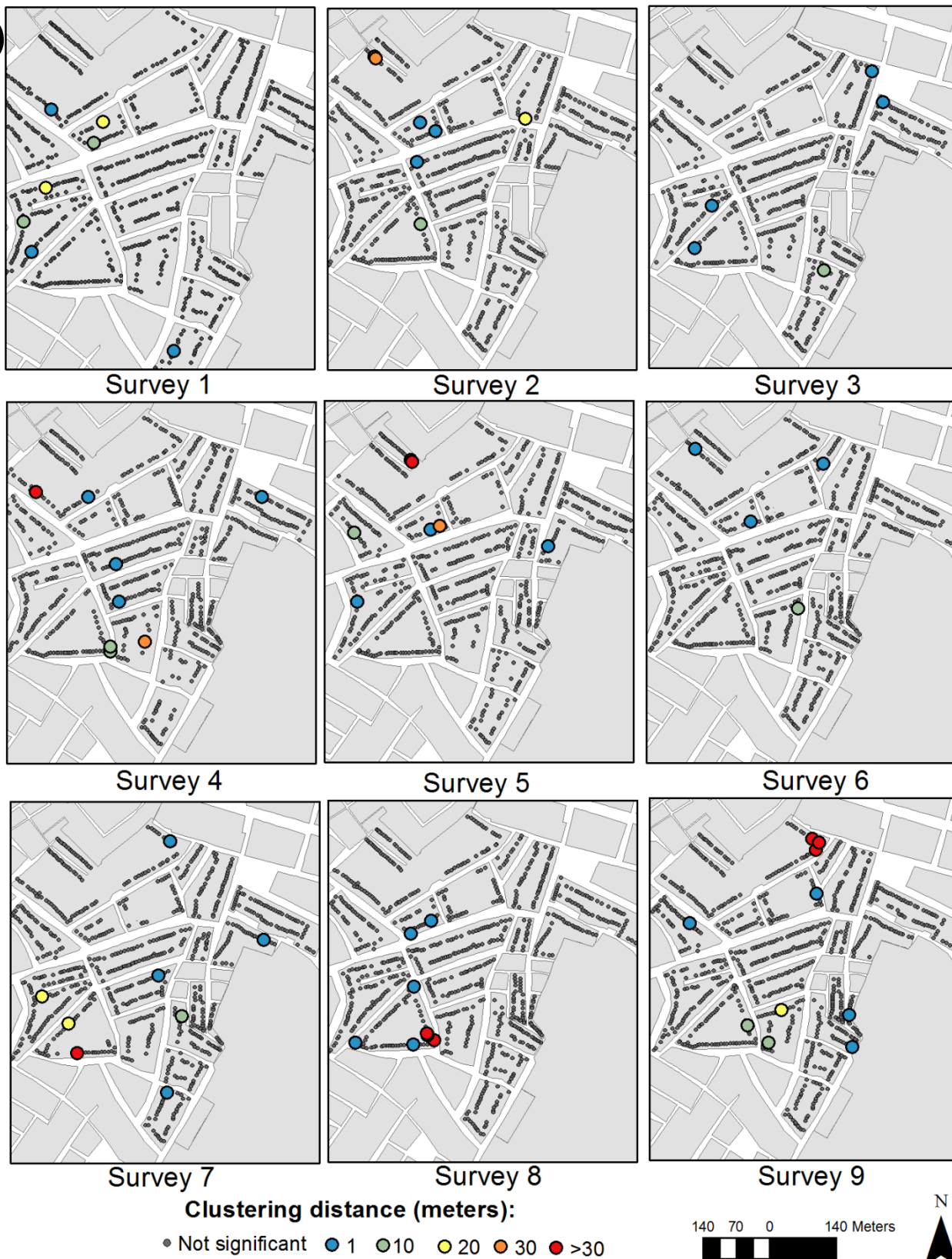

Supplement: Figure S4 — Distance up to which adult male and female Ae. aegypti abundance clustered. Maps show the results of the Gi* tests by entomologic survey for Maynas (A) and Tupac Amaru (B) neighborhoods. Households for which no clusters were detected were labeled as NS (not significant). (PDF) [file pntd.0003038.s004.pdf]

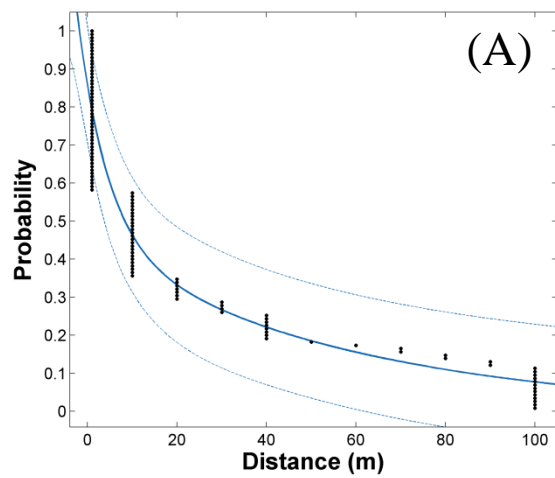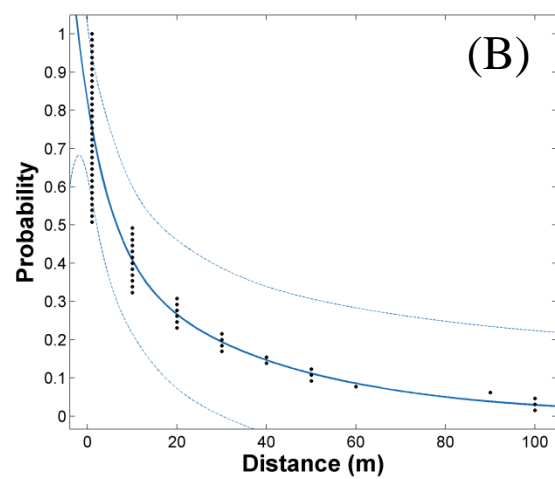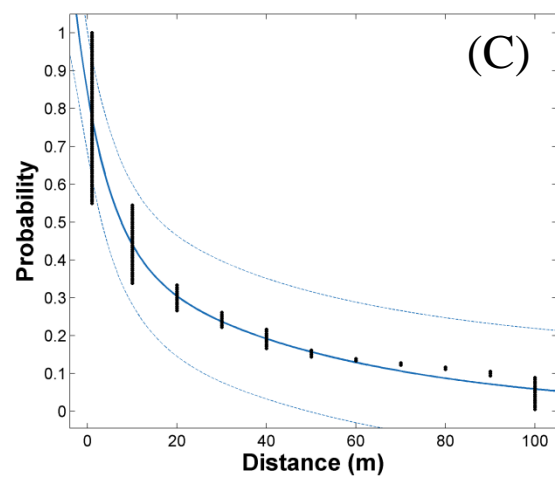

Supplement: Figure S5 — Probability of finding spatially correlated adult male or female Ae. aegypti populations at increasing distances from a household. Panels show data from 9 entomologic surveys performed in (A) Maynas, (B) Tupac Amaru and (C) both neighborhoods combined. Solid line shows exponential fit results together with its 95% confidence interval (dotted line). (PDF) [file pntd.0003038.s005.pdf]
